# Ranking nodes in growing networks: When PageRank fails

**DOI:** 10.1038/srep16181

**Published:** 2015-11-10

**Authors:** Manuel Sebastian Mariani, Matúš Medo, Yi-Cheng Zhang

**Affiliations:** 1 Department of Physics, University of Fribourg, 1700 Fribourg, Switzerland; 2 Institute of Fundamental and Frontier Sciences, UESTC, Chengdu 610054, China

## Abstract

PageRank is arguably the most popular ranking algorithm which is being applied in real systems ranging from information to biological and infrastructure networks. Despite its outstanding popularity and broad use in different areas of science, the relation between the algorithm’s efficacy and properties of the network on which it acts has not yet been fully understood. We study here PageRank’s performance on a network model supported by real data, and show that realistic temporal effects make PageRank fail in individuating the most valuable nodes for a broad range of model parameters. Results on real data are in qualitative agreement with our model-based findings. This failure of PageRank reveals that the static approach to information filtering is inappropriate for a broad class of growing systems, and suggest that time-dependent algorithms that are based on the temporal linking patterns of these systems are needed to better rank the nodes.

With the amount of available information constantly growing due to the widespread usage of computers and the Internet, network-driven information filtering tools such as ranking algorithms^1^,^2^ and recommender systems^3^ attract attention of researchers from various fields. PageRank, one of the most popular ranking algorithms, has been originally devised to rank web sites in search engine results^4^. The algorithm acts on unipartite directed networks and builds on the circular idea *“A node is important if it is pointed by other important nodes”*. The essential role that PageRank plays in the Google search algorithm has stimulated extensive research of its properties^5^ and relations to previous ranking techniques^6^. PageRank has been applied far beyond its original scope: in ranking of scholarly papers^7^, authors^8^,^9^ and journals^10^, ranking of images in search^11^, ranking of urban roads according to traffic flow^12^, measuring the importance of biochemical reactions in the metabolic network^13^, for example. The algorithm’s remarkable stability properties^5^,^14^ make it a suitable candidate to rank nodes in noisy networks such as the World Wide Web (WWW) and the protein interaction networks, where the information is often not completely reliable. Variants of PageRank include Eigentrust which computes trust values in distributed peer-to-peer systems^15^, LeaderRank which computes influence of users in social networks^16^, and CiteRank which uses a model of citation network traffic to compute the importance of scientific papers^17^, among others; variants of PageRank have been also applied to bipartite networks^18^,^19^,^20^ and multilayer networks^21^.

The widespread usage of PageRank motivates us to ask: when is the algorithm effective in ranking nodes according to their quality? Are there circumstances under which the algorithm is doomed to fail? Answering these questions is of primary importance to foster our understanding of the ranking algorithm, which is a problem of practical significance given the influence of ranking-based tools such as search engines and recommendation systems on many aspects of our society, from marketing to politics^22^,^23^,^24^,^25^. While previous research has already studied the rankings produced by PageRank for different topological properties of the input networks^14^, the evaluation of the algorithm on networks that evolve in time remains a largely unexplored field. The main aim of this work is to fill this gap and demonstrate the shortcomings of the algorithm when applied to growing networks exhibiting temporal effects. To this end, we use a growing directed network model with preferential attachment and relevance^26^ which generalizes the classical preferential attachment introduced in^27^. This model (hereafter the Relevance Model, RM) has been shown by maximum likelihood analysis to be the preferential attachment model that best explains the linking patterns in real information systems^28^ and has been used to model real information systems, such as the WWW^29^, citation networks^30^, online networks^28^, and even technological networks, such as the network of Internet autonomous systems^31^.

In the RM, three essential elements rule the competition among nodes for incoming links: preferential attachment, fitness and temporal decay. Preferential attachment is a well-established mechanism that has been observed in a wide range of real systems (see^32^,^33^ for a review). *Fitness* is a quality parameter assigned to each node that quantifies the node’s inherent competence in attracting new incoming links^34^; all other things being equal, in a competitive environment high-fitness nodes are suitable for success in the system and are likely to become eventually popular, whereas low fitness nodes tend to remain little known^29^. Node fitness is modulated with a time-decaying function which gives rise to the so-called node relevance^26^: a node of high-fitness thus initially has high relevance and potentially attracts many links but this relevance eventually vanishes and the node ceases to attract new links. Fitness and relevance discount all system-dependent intangible and subjective factors that determine node’s quality, quantify how much a node is attractive to a given system and can be estimated on real data by different techniques^26^,^28^,^29^,^30^. In our model, each node is further endowed with an activity parameter which represents the rate at which the node creates new outgoing links; activity too is modulated with time. We use the model to produce artificial data and compare the ranking of nodes by their indegree (i.e., the number of incoming links) and PageRank score with the node ranking by their fitness values. We find that when model parameters for the temporal decay of relevance and activity substantially differ from each other, the redistribution of PageRank scores is biased towards old or recent nodes, respectively (depending on which decay is faster). In addition, when PageRank is temporally biased in either way, indegree markedly outperforms it in ranking nodes by their fitness. These results are confirmed on a modified model, so-called Extended Fitness Model, where high-fitness nodes preferentially attach to other high-fitness nodes, whereas low-fitness nodes preferentially attach to popular nodes. While in this model PageRank can significantly outperform indegree in reproducing the ranking of nodes by their fitness for some model parameters, extensive parameter regions where the algorithm fails and performs worse than indegree are still present.

We finally apply PageRank on two real datasets, the social network of Digg.com users and the network of citations between American Physical Society (APS) scientific articles, and compare the rankings of nodes by their indegree and PageRank score with the node ranking by their total relevance which is a real-data estimate for fitness. We find that while PageRank score is highly correlated with indegree in social network data and the two metrics have similar performance, PageRank is markedly outperformed by indegree in citation data. These findings strongly discourage the use of PageRank in systems where strong temporal patterns exist, like citation networks.

## Results

### Relevance Model (RM)

In the RM, when a node *j* creates a new link at time *t*, the probability 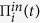 that it chooses node *i* as the target is assumed to be


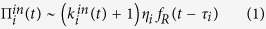


where 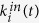 is the current indegree of node *i*, 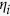 is its fitness and 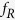 is a function of the node’s age (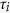 is the time at which node 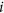 enters the system). The product 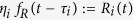 represents the *relevance* of node *i* at time *t*^26^,^28^. We assume that 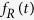 decays monotonously and thus mimics real situations where nodes lose relevance over time. Previous studies of the RM^26^,^30^ have focused on scientific citation networks which are tree-like because nodes create outgoing links only in the moment when they enter the system – the links are thus always directed back in time. We consider a general situation where nodes continue being active, create outgoing links continually, and the resulting network thus contains loops which are common in many real systems, such as the WWW, for example. We use the activity potential approach introduced in^35^ and assign to each node *i* an activity parameter *A*_*i*_. At each simulation step, a new node is created and connected to an existing node. In addition, 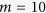 existing nodes are sequentially chosen and create one link each (see the Methods section for all simulation details). The *m* nodes that are active at time *t* are chosen with the probability


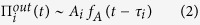


where 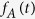 is a monotonously decaying function of time. A broad distribution of the activity parameter *A* allows us to reproduce broad outdegree distributions typically found in real networks^33^ without resorting to preferential linking mechanisms for outgoing links.

### Decay of empirical relevance and activity in real data

We now analyze real data to validate the hypothesis of relevance and activity decay. We refrain from maximum likelihood analysis^28^ because of its computational complexity. Instead, we follow a simpler procedure: following^26^, we define the empirical relevance 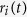 of node *i* at time *t* as


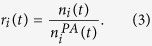


Here 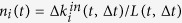 is the ratio between the number 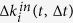 of incoming links received by node *i* in a suitable time window 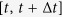 and the total number 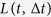 of links created within the same time window, whereas 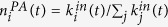 is the expected value of 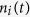 according to preferential attachment alone. Empirical relevance 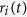 larger or smaller than one means that node *i* at time *t* outperforms or underperforms, respectively, with respect to its preferential attachment weight 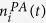 in the competition for incoming links.

The hypothesis of time-dependent and heterogeneous relevance has already been validated in the APS scientific citation network^26^. Here we further analyze the APS dataset, described in the Methods section, finding (Fig. S2) that the decay of relevance is well reproduced by a power law function (see the Supplementary Note S2 for detailed results). Moreover, we validate the hypothesis of relevance and activity time decay in a very different system, the Digg.com social network of users, where a directed link between two users means that one user follows the other (see the Methods section for the description of the dataset). We find (Fig. S1) that relevance decays also in this dataset. Based on^35^, we define the empirical activity 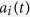 of node *i* at time *t* as the ratio between the number of outgoing links created by node *i* in a suitable time window 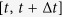 and the total number of links created within the same time window. We find (Fig. S1) that also activity decays with time, and activity decay is slower than relevance decay (see Supplementary Note S1 for details).

### Results of numerical simulation with the RM

For the sake of generality, we consider both exponential and power-law decay functions 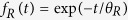, 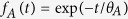 and 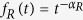, 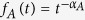, respectively. Our main goal now is to study the dependence of PageRank performance on model parameters 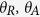 and 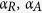, respectively. We refer to the Methods section for the mathematical definition of PageRank and details about the choice of fitness and activity distributions in simulations.

**Figure 1:**
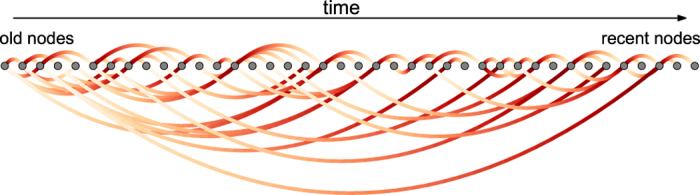
Illustration of a network produced with the RM for fast aging of relevance and constant activity. In each step, a new node is introduced and connected to an existing node (arcs above the row of nodes). In addition, a randomly chosen node becomes active and connects to an existing node (arcs below). The target node is chosen by Eq. (1) in both cases (see Supplementary Note S5 for model parameters). The orange and red part of the each link mark the initial and target node, respectively. Note that while old nodes point to nodes of every age thanks to constant activity, recent nodes never point to the old nodes due to the decay of relevance. This asymmetry results in PageRank scores biased towards recent nodes.

A good ranking algorithm is expected to produce an unbiased ranking where both recent and old nodes have the same chance to appear at the top. In growing networks with temporal effects, PageRank can fail to achieve this. To explain the origin of this failure, we consider two extreme situations: relevance decay which is very fast and slow, respectively, with respect to activity decay. When relevance decay is slow (or absent, as in the original fitness model^34^), recent nodes receive few links because their weight in preferential attachment is much smaller than the weight of all nodes that have already accumulated many links (this manifests itself in the network’s strong dependence on the initial configuration^36^). PageRank as well as indegree are therefore strongly biased towards old nodes. When relevance decay is fast, preferential attachment is compensated by a quick decay of relevance and therefore recent nodes can reach high indegree. However, there is now an essential asymmetry in the system which relates to outgoing links: while recent nodes mostly point to other recent nodes because of relevance decay, old nodes point to nodes of every age because they remain active during the whole system’s lifetime (see Fig. 1 for an illustration). PageRank is consequently biased towards recent nodes: while a random surfer at an old node is likely to jump to a recent node, the converse is not true; recent nodes effectively act as an attractor.

**Figure 2:**
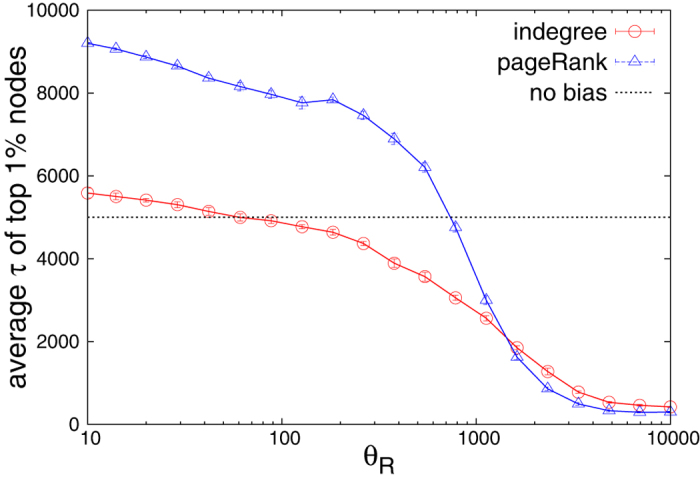
PageRank time bias. We show here the average entrance time 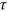 of the top 1% nodes of the node ranking by indegree and PageRank, respectively, as a function of the relevance decay parameter 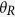. Networks of 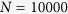 nodes are grown with the RM with slow decay of activity (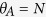). Two limits of PageRank bias are visible: (1) When the decay of relevance is fast 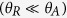, a large number of top nodes are recent as a consequence of the network structure demonstrated in Fig. 1; (2) When the decay of relevance is slow (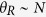), top nodes are old because the old nodes can be pointed by nodes of every age. While the latter bias is common to PageRank and indegree, the former bias is specific to PageRank because of its network nature.

Figure 2 shows a transition between the two extreme cases for artificial data produced by the RM with exponential relevance decay and exponentially distributed fitness. When the decay of relevance is slow (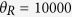), there are only old nodes at the top 1% positions of the rankings by PageRank score and indegree. When the decay of relevance is fast (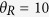), recent nodes occupy the majority of the top 1% positions in the ranking by PageRank score. By contrast, the ranking by indegree is essentially unbiased in this limit as the average entrance time 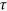 of the top-1% nodes is close to 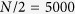 which corresponds to the absence of time bias.

**Figure 3:**
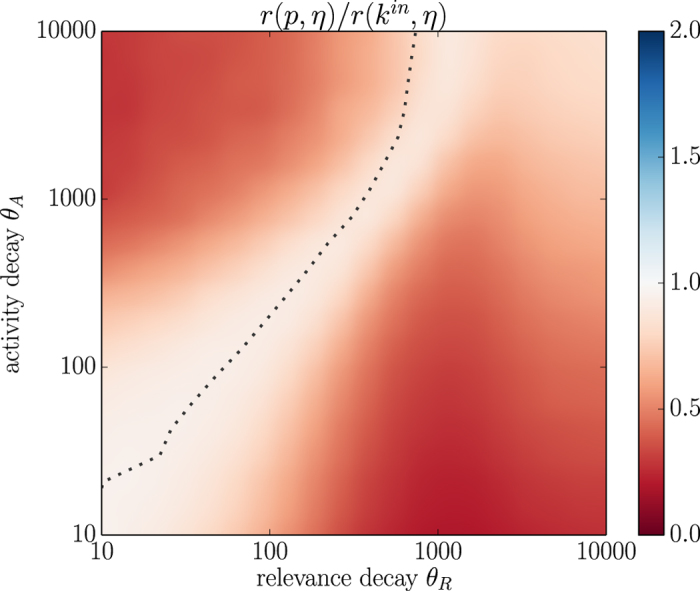
A comparison of performance of PageRank and indegree in the RM data (*N* = 10,000. *ρ*(*η*) = exp(−*η*). The heatmap shows the ratio 
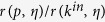
. The black dotted line represents the contour along which PageRank is not temporally biased (see Fig. S6, left). The upward bending of this contour is a finite-size effect.

We discuss now the implication of PageRank’s time bias on the algorithm’s ability to rank nodes by fitness. In the following, we denote by 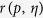 the Pearson’s correlation between the PageRank scores *p* and the fitness values 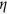, and we denote by 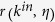 the Pearson’s correlation between node indegree and fitness. Figure 3 shows the performance ratio 
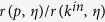
 in the 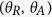 plane. Since 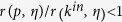 everywhere, we find that PageRank yields no improvement with respect to indegree in ranking nodes by fitness. This is because while the PageRank algorithm assumes that important nodes point to other important nodes, this feature is absent in the RM where all nodes are driven by the same mechanism, Eq. (1), when choosing their connections. As a result, PageRank does best in comparison with indegree along the 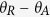 diagonal where PageRank is not temporally biased and 
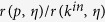
 becomes close to, albeit always strictly lower than, one. When moving away from this diagonal, PageRank score has temporal bias towards recent or old nodes (Fig. S6), its correlation with indegree (Fig. S7) and fitness (Fig. S8) decrease, and it reproduces fitness substantially worse than indegree (red areas in Fig. 3). Qualitatively similar behavior is found for the RM with uniformly distributed fitness (Fig. S9), power-law decay of relevance and activity (Fig. S10), accelerated growth rate (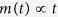 instead of 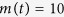, Fig. S12). The same is true when the ranking quality is measured by the precision metric 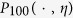, (defined as the number of fitness top-100 nodes placed in the top 100 of the ranking produced by an algorithm), instead of the linear correlation coefficient (Fig. S11). This shows that our findings are robust and do not require a specific model setting.

### An extended model based on fitness

To demonstrate that PageRank’s under-performance with respect to indegree is a general feature, we now proceed to a different model for artificial data which is more compatible with PageRank’s basic idea that a node is important if it is pointed by other important nodes. In this model (hereafter Extended Fitness Model, EFM), high- and low-fitness nodes differ not only in their ability to attract new incoming links, but also in their sensitivity to the fitness of the other nodes when choosing their outgoing connections. High-fitness nodes are highly attractive to new incoming links as well as highly sensitive to fitness of the others when choosing their outgoing connections. Low-fitness nodes are basically insensitive to fitness and choose their target nodes mostly by current popularity amended by aging. High-fitness nodes are then more likely to be pointed by other high-fitness nodes than low-fitness nodes (see Fig. S5) which agrees with the basic premise of PageRank: important nodes are pointed by other important nodes. We therefore expect PageRank to outperform indegree in ranking the nodes by fitness. The model assumes that the probability 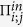 that a link created by node *j* at time *t* ends in node *i* has the form


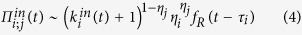


where node fitness 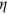 is now constrained to the range [0, 1] to prevent a negative exponent 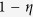 in the first term. We stress that the probability 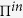 depends not only on the fitness 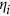 of the target node, but also on the fitness 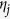 of the node *j* that creates the outgoing link, which is a new element with respect to the RM. A similar model has been used to model user-item networks in^37^. We assume that a small number *H* of nodes have high fitness 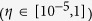 and the remaining 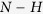 nodes have low fitness (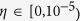, see the Methods section for details).

**Figure 4:**
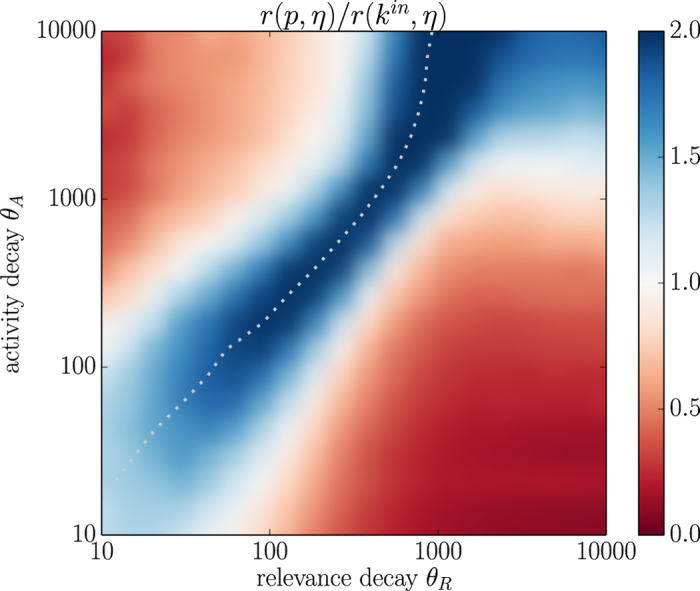
A comparison of PageRank and indegree correlation with fitness in the EFM data (*N* = 10,000, *H* = 250). The heatmap shows the ratio 
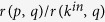
. The white dotted line represents the contour where PageRank is not temporally biased (see Fig. S6, right).

Figure 4 shows the results obtained with the EFM. The correlation coefficient 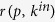 (Fig. S7, right) and the average age of top 1% nodes (Fig. S6, right) have qualitatively the same behavior as for the RM which indicates that the behaviour of these quantities as a function of model’s temporal parameters is universal and independent of the exact growth rule. The model is favorable to PageRank and indeed, the algorithm now can significantly outperform indegree in terms of the correlation between fitness and node score when PageRank is not temporally biased (blue area in Fig. 4). Nevertheless, PageRank still underperforms indegree in two extensive regions of the parameter plane 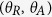. As for the RM, these two regions correspond to the cases where activity and relevance decay timescales substantially differ. These results are again confirmed by using power-law aging instead of exponential (Fig. S10) and the precision metrics instead of the correlation coefficient (Fig. S11). Note that we introduced here the EFM to show that PageRank’s bias occurs also in a setting favorable to the algorithm; while it seems plausible that some nodes are more sensitive to fitness than others when making connections, we leave real data validation of the EFM for future research.

### Comparing indegree and PageRank: results in real networks.

**Figure 5:**
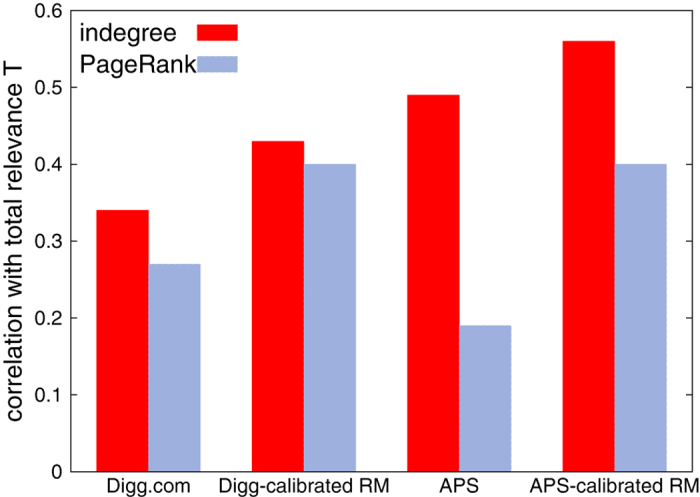
A comparison of PageRank and indegree correlation with total relevance in real data and in calibrated simulations with the RM. PageRank is outperformed by indegree in both datasets (and in the corresponding calibrated simulations). In the Digg.com social network, the fitted relevance and activity power-law decay exponents are not far from the parameter region where PageRank is maximally correlated with indegree in numerical simulations with the RM with power-law decay (see Fig. S10), and PageRank’s and indegree’s correlation with total relevance are close to each other. By contrast, in the APS dataset activity decays immediately, whereas relevance decays progressively (see Fig. S2); as a consequence, PageRank is strongly biased towards old nodes (see Fig. S3) and is outperformed by indegree by a factor 2.58 [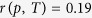 whereas 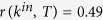]. We refer to the Supplementary Note S3 for details about the simulation calibration on real data and to the Supplementary Note S4 for details on the computation of empirical relevance in real and artificial data.

Algorithm evaluation in real data is made difficult by several factors. In general, it is impossible to objectively evaluate node importance in a system because it depends on many intangible and subjective elements^6^. To assess the performance of ranking algorithms on real data, we compare node score with *total relevance* 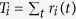 which is an estimate of node fitness (see ref. 26 and the Supplementary Note S4). Results on real data and the corresponding calibrated simulations with the RM are reported in Fig. 5. Our calibration procedure for simulations focuses on temporal decay of relevance and activity and is described in detail in the Supplementary Note S3; more accurate calibration is possible but goes beyond the scope of our work. Uncertainty of these results estimated by sample-to-sample fluctuations and non-parametric bootstrap^38^ for model and real data, respectively, is of the order of 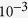 which is negligible in comparison with the observed differences between PageRank and indegree (see Supplementary Note S6).

In the Digg.com social network, the empirircal relevance and activity power-law decay exponents are not far from the parameter region where PageRank scores are maximally correlated with indegree in the simulations with the RM with power-law decay (see Fig. S10), which is in qualitative agreement with the observed high value of correlation between PageRank and indegree in the dataset (
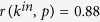
); PageRank is outperformed by indegree in ranking nodes by their total relevance but the performances of the two metrics are relatively close to each other (see Fig. 5).

In citation data, where the use of PageRank and other algorithms inspired by PageRank has been much studied^7^,^17^,^39^, activity and relevance decays necessarily mismatch: relevance progressively decays with time^26^, whereas activity decays immediately. In the APS dataset we find that PageRank is significantly biased towards old nodes (Fig. S3): this is because old papers can be pointed by papers of every age, while recent papers are pointed only by recent papers. This is the opposite time bias than that depicted in Fig. 1. Moreover, we find that PageRank and indegree are weakly correlated [
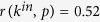
], and indegree is remarkably better correlated with total relevance than PageRank (see Fig. 5). These findings are consistent with the outcomes of a calibrated numerical simulation with the RM (see Fig. 5), where all outgoing links of a node are created when the node enters the system and the outdegree distribution is exponential as in the APS dataset (Fig. S4), see the Supplementary Note S3 for details about simulations calibrated on real data). Note that the age distribution of top nodes in indegree and PageRank ranking in the APS real network and the artificial network generated by the corresponding calibrated simulation have the same qualitative shape (Fig. S3). This confirms that our simulation calibration on real data, which is based only on the temporal patterns of the system, qualitatively captures the temporal bias of PageRank.

We conclude this paragraph with a consideration on total relevance *T*. Motivated by the high correlation 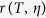 between node total relevance and fitness found in the calibrated simulations (see Supplementary Note S4), in this work we use total relevance *T* as a proxy for node fitness in the real data. In the RM, we find that node total relevance outperforms indegree and PageRank in ranking nodes by fitness for a broad range of model parameters (Fig. S13). By contrast, the parameter region where total relevance outperforms indegree and PageRank is smaller in data produced with the EFM (Fig. S14). We leave for future research detailed investigation of how the performance of total relevance in ranking nodes by fitness depends on the assumptions and parameters of the underlying model. These findings might also motivate future study of the ranking of nodes by their total relevance in real data that are well-described by the RM.

## Discussion

To summarize, our numerical simulations indicate that the mismatch between the timescales of relevance and activity decay makes PageRank scores biased towards recent nodes (when the decay of relevance is faster) or old nodes (when the decay of activity is faster). This temporal bias reduces PageRank’s capability to rank nodes by fitness and causes it to underperform in comparison with the elementary ranking of nodes by indegree in the RM which is to our best knowledge the most accurate model for describing growing information networks^28^,^30^. Our findings are robust with respect to changes in the functional form of the time-decay function, in the distribution of fitness among the nodes, and in the metric used to evaluate the ability of an algorithm to rank nodes by their fitness. We also studied a model (the EFM) that provides a favorable setting for PageRank performance; PageRank can outperform indegree on the data produced by this model, but fails again when the two timescales mismatch. Moreover, we find indications of the influence of temporal patterns on PageRank’s performance also in real data. In citation data, PageRank is excessively biased towards old nodes and, as a consequence, is clearly outperformed by indegree in ranking nodes by their total relevance which is an estimate of node fitness (see ref. 26 and the Supplementary Note S4). By contrast, indegree and PageRank perform similarly in social network data where there is not a sharp mismatch between activity and relevance timescales. The results of real data analysis are in agreement with our model-based finding that PageRank can only perform well if the two system’s timescales (of activity and relevance decay, respectively) are of similar magnitude.

The methods developed and used in this article are general and can be applied to any growing directed network where nodes compete for incoming links and where preferential attachment and temporal effects influence the linking patterns, which includes a wide class of real networks. To diagnose whether a growing directed network is or not suitable for the application of PageRank, one can fit the empirical relevance decay and activity decay timescales on the data and run a corresponding calibrated simulation which reveals whether PageRank is or not able to rank nodes according to their fitness. We have not attempted to study how our findings are affected by further real-world phenomena, such as link deletion^29^, popularity^40^ and activity bursts^41^, among others. Link time stamps are crucial for our analysis; in all the datasets where they are not reported, we cannot compute neither node relevance 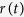 nor node activity 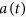 which exclude these systems from the range of applicability of our analysis. We also stress that the framework introduced in this work is not applicable to undirected networks, such as collaboration networks, scholar co-citation networks and road networks, among others. In undirected networks indeed there is no distinction between incoming and outgoing links and, as a consequence, relevance and activity cannot be defined as two separate node properties. The model-based evaluation of PageRank’s performance in networks without time information and undirected networks is certainly an interesting and largely unexplored problem but goes beyond the scope of this work.

The shortcoming of PageRank due to temporal effects is particularly worrying for applications of the algorithm to scientific citation data^10^,^39^,^42^. While PageRank can find old valuable papers underestimated by indegree^7^, the algorithm is biased towards old nodes and as a consequence is outperformed by indegree in ranking papers by importance, which strongly discourage the use of the algorithm to rank scientific papers. In this context, the need for including temporal effects in the algorithm has already been stressed in^17^,^39^; the model-based approach introduced in this article leads us to the same conclusion. How to best include the temporal dimension in ranking scientific publications remains an open issue. One could consider a self-consistent algorithm that takes time into account, such as CiteRank^17^, or resort to fitness estimates, such as total relevance or maximum likelihood estimates^28^; the model-based approach introduced in this article provides a simple yet effective method – the comparison of node scores with intrinsic fitness in calibrated simulations – which could be used to establish which algorithm is more suitable for a given system. Our findings also bring new insights into the study of the relation between node indegree and PageRank. Previous studies^25^,^43^ established a linear relationship between node degree and the average PageRank score for uncorrelated networks, and considered any deviations from this behavior as fluctuations. We find that for a broad range of network parameters, much of these apparent fluctuations are in fact trends caused by the interplay between the network’s temporal features and the PageRank algorithm.

Our model-based evaluation of ranking algorithm is applicable also to the WWW. There is general agreement in recognizing the importance of PageRank in the success of Google’s search engine^6^,^44^, yet it remains unclear which properties make the Web a suitable network where to apply the algorithm. While ref. 14 emphasizes the role of the scale-free topology of the Web on PageRank’s success, our findings stress the importance of temporal patterns in determining the success or failure of PageRank. Further data analysis on Web data could reveal whether relevance and activity decay timescales are of similar magnitude in the WWW which would imply maximal correlation between PageRank score and node fitness, and thus provide a further explanation of PageRank’s success in this system.

In conclusion, PageRank, despite its popularity and robustness, can fail and thus it should not be used without carefully considering the temporal properties of the system to which it is to be applied. The connection between PageRank’s failure and the temporal features of the analyzed networks indicates that the main reason for the reported failure is the static nature of the algorithm. We believe that a well-grounded ranking algorithm should be built on the temporal linking patterns of the system where it is intended to be applied and the dependence of its performance on system features should be exhaustively studied in model data where system’s structural and temporal properties can be modified simply by changing model parameters. We believe that the model-based theoretical evaluation of ranking algorithms developed in this work will open the door to systematic performance evaluation of algorithms in evolving systems, deepen our understanding of their limitations, and lead to the introduction of new improved algorithms.

## Methods

### Digg.com dataset

Digg.com had been an online social news aggregator from December 2004 to July 2012. Digg.com users were allowed to submit and vote (“digg”) stories. Interaction between users took place through comments and messages (see^45^ for a detailed description of the website). We studied the social network of users where nodes represent the users and a link from node *i* to node *j* means that user *i* is a follower of user *j*. The complete dataset in our possession covers the period from 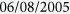 to 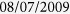. We analyzed a 3-years subset running from 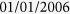 to 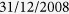. The subset consists of 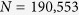 nodes and 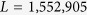 links.

### APS dataset

The APS (American Physical Society) dataset in our possession spans from year 1893 until 2009 and contains 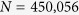 nodes (papers) and 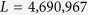 directed links (citations) between them. This dataset has been used in^26^ to validate the hypothesis of heterogeneous and decaying relevance.

### PageRank

In a directed monopartite network composed of *N* nodes, the vector of PageRank scores {*p*_*i*_} can be found as the stationary solution of the following set of recursive linear equations


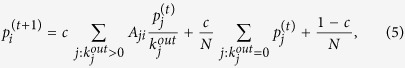


where *A* is the network’s adjacency matrix (*A*_*ji*_ is one if node *j* points to node *i* and zero otherwise), 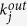 is the outdegree of node *j*, *c* is the teleportation parameter, and *t* is the iteration number^4^. Eq. (5) represents the master equation of a diffusion process on the network, which converges to a unique stationary state independently of the initial condition^46^. The PageRank score *p*_*i*_ of node *i* can be interpreted as the average fraction of time spent on node *i* by a random walker who with probability *c* follows the network’s links and with probability 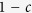 teleports to a random node. We set 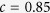 which is the usual choice in practice^46^. Iterations are stopped when the modulus distance between the vectors of scores at two consecutive iterations becomes smaller than 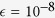 46.

### Simulation details

We use the artificial models (RM and EFM) to build monopartite directed networks composed of 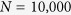 nodes. We start from a configuration with two nodes, node 0 and node 1, and a link from node 1 to node 0. At each simulation step *t*, we add a new node *t* to the system and connect it to an already existing node. The target node is chosen according to the attachment rule (1) (RM) or (4) (EFM). If 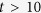, we also sequentially add 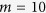 links between the existing nodes. Their initial nodes are chosen according to the activity rule (2); the target nodes follow again Eqs (1) or (4), respectively. The creation of multiple links between a pair of nodes and self-loops are prohibited. Unless stated otherwise, results are averages over 6 realizations of the model. Error bars In Fig. 2 represent the standard error of the mean which is generally small. The same is true for Figs 3 and 4 where only the average values are displayed.

### Fitness and activity distributions in the RM

When relevance decay is sufficiently fast to allow the normalization factor 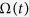 of 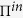 to converge within the simulation time scale^26^, the average final indegree of a node in the RM depends exponentially on node fitness. Consequently, different fitness distributions yield different indegree distributions^26^,^34^. We use both exponential and uniform fitness distribution in our simulations; results for the latter are shown in Fig. S9. The outdegree distribution is only determined by the activity distribution 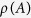 (see Supplementary Note S7 for basic analytical results). In our simulations we use 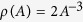 for 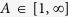 everywhere except for the calibrated APS data simulation where all outgoing links of a node are created when the node enters the system and we use 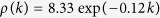 for 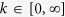 as the outdegree distribution, as found in the APS data (see Fig. S4).

### Fitness and activity distribution in the EFM

We choose here a fitness distribution that aims to emphasize the difference between the linking pattern of high- and low- fitness nodes without trying to reproduce structural features of real data. The set of fitness values consists of 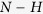 equidistant values within the interval 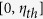 (low-fitness nodes) and *H* equidistant values from the range 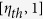 (high-fitness nodes). These values are then bijectively assigned to the network’s *N* nodes at random. We set a small value of the threshold 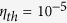 which implies that the low-fitness nodes are essentially insensitive to node fitness, while the high-fitness nodes range from little fitness-sensitive nodes to nodes almost unaffected by popularity and mainly driven by fitness (when 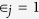, we have 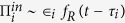). We run simulations with 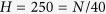 for Fig. 4. this value is small in order to amplify the advantage of high-fitness nodes in connecting to other high-fitness nodes (see Fig. S5). As in the RM, we use 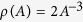 for 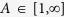 to generate the node activity values.

## Additional Information

**How to cite this article**: Mariani, M. S. *et al.* Ranking nodes in growing networks: When PageRank fails. *Sci. Rep.* **5**, 16181; doi: 10.1038/srep16181 (2015).

## Supplementary Material

Supplementary Information
