# Supplementary material for: Ranking nodes in growing networks: When PageRank fails
Source: Sci Rep. 2015 Nov 10;5:16181. doi: 10.1038/srep16181 (PMC4639772; doi:10.1038/srep16181)
Supplement: Supplementary Information [file srep16181-s1.pdf]

Ranking nodes in growing networks: When PageRank fails – Supplementary Information

Manuel Sebastian Mariani, Matúš Medo, and Yi-Cheng Zhang

Contents

S1 Analysis of empirical relevance and activity in the Digg.com dataset 2

S2 Analysis of empirical relevance in the APS dataset 2

S3 Simulations calibrated on real data 3

S4 Measuring empirical relevance in real and artificial data 3

S5 Production of Figure 1 in the main text 3

S6 Assessing the uncertainty of results 3

S7 Relation between outdegree and activity 4

S8 Supplementary figures: Analysis of real data 5

S9 Supplementary figures: Numerical simulations 7

List of Figures

S1 Temporal decay of the average relevance $r(t)$ and activity $a(t)$ in Digg.com social network 5
S2 Temporal decay of the average relevance $r(t)$ in the APS dataset 5
S3 Age distribution of the top 1% nodes in the ranking (APS data and the corresponding calibrated simulation) 6
S4 Outdegree distribution in the APS dataset 6
S5 Linking pattern in the extended fitness model (EFM) 7
S6 Average birth time τ of the top 1% of nodes as ranked by PageRank 8
S7 Indegree-PageRank correlation in the RM and EFM 8
S8 Fitness-PageRank correlation in the RM and EFM 9
S9 Comparison between PageRank and indegree in the RM with uniform fitness distribution 9
S10 Comparison between PageRank and indegree in the RM and EFM with power-law aging 10
S11 Comparison between PageRank and indegree in the RM and EFM using precision . . 11
S12 Comparison between PageRank and indegree in the RM with accelerated growth . . . 11
S13 Comparison of total relevance with indegree (left) and PageRank (right) in the RM . . 12
S14 Comparison of total relevance with indegree (left) and PageRank (right) in the EFM . 12

Relevance decay

Node group	C	α	D
$k^{in} > 100$	61.31	1.09	0.50
$k^{in} \in [10, 100]$	25.00	0.99	0.46
$k^{in} < 10$	1.85	0.76	0.12

Activity decay

Node group	C	α	D
$k^{out} > 100$	$1.1 \cdot 10^{-3}$	0.38	$-6.0 \cdot 10^{-5}$
$k^{out} \in [10, 100]$	$2.2 \cdot 10^{-4}$	0.44	$-1.86 \cdot 10^{-5}$
$k^{out} < 10$	$1.4 \cdot 10^{-5}$	0.44	$-1.38 \cdot 10^{-6}$

Table 1: Parameter estimation results for the average relevance and activity decay in the Digg.com data.

Relevance decay

Node group	C	α	D
$k^{in} > 100$	217.5	1.40	0.19
$k^{in} \in [10, 100]$	250.8	1.51	0
$k^{in} < 10$	177.2	1.51	0

Table 2: Parameter estimation results for the average relevance decay in the APS data.

Supplementary notes

S1 Analysis of empirical relevance and activity in the Digg.com dataset

As explained in the main text, the empirical relevance $r_i(t)$ of node i at time t is defined as

$$r_i(t) = \frac{n_i(t)}{n_i^{PA}(t)} \tag{S1}$$

where $n_i(t) = \Delta k_i^{in}(t, \Delta t)/L(t, \Delta t)$ is the ratio between the number $\Delta k_i^{in}(t, \Delta t)$ of incoming links received by node i in a suitably chosen time window $[t, t + \Delta t)$ and the total number $L(t, \Delta t)$ of links created within the same time window, whereas $n_i^{PA}(t) = k_i^{in}(t)/\sum_j k_j^{in}(t)$ is the expected value of $n_i(t)$ according to preferential attachment alone [1]. The activity $a_i(t)$ of node i at time t has been defined in [2] as

$$a_i(t) = \frac{\Delta k_i^{out}(t, \Delta t)}{L(t, \Delta t)} \tag{S2}$$

where $\Delta k_i^{out}(t, \Delta t)$ is the number of outgoing links created by node i in the time window $[t, t + \Delta t)$. We use $\Delta t = 1$ week to compute relevance and activity in the Digg.com dataset.

Figure S1 shows the average temporal decay of relevance and activity in the Digg.com dataset (see MM section of the main text for a description of the dataset). We fit both decay profiles with a power-law function $f(t) = C\,t^{-\alpha} + D$ with three parameters (C, α, D) using the least-squares method. While other and perhaps more accurate patameter estimation procedures exist, the present results are sufficient for our analysis. We only consider the first 100 weeks after the first link received/created by the respective node. Parameter estimates are summarized in Table 1 separately for nodes of high, medium and low in-degree and out-degree, respectively. One may note here that activity decays slower (with a lower exponent) than relevance.

S2 Analysis of empirical relevance in the APS dataset

We use $\Delta t = 91$ days to calculate empirical relevance of nodes in the APS dataset (see MM section of the main text for a description of the dataset). Figure S2 shows the average relevance decay in the APS dataset; it is analogous to Figure 1 in [1]. Similarly as for the Digg.com dataset, we fit the results with the power law dependence $f(t) = C\,t^{-\alpha} + D$. To avoid the non-monotonous initial behavior of relevance (which is due to, for example, the time needed to carry out and publish research building on a given paper), we ignore the first 5 years (10 years for low indegree nodes) after publication. The estimation results are reported in Table 2.

S3 Simulations calibrated on real data

When calibrating the numerical simulations on the Digg.com and APS datasets, we focus only on the datasets' temporal patterns that constitute the main motivation of our study and are the principal reason for the reported failure of PageRank. While more accurate calibration of models to the real data is possible, we do not find it necessary because our calibrated simulations capture some basic temporal patterns of indegree and PageRank scores (see Figure S3).

The artificial dataset calibrated on the Digg.com data is grown using the relevance model (RM) with $\rho(\eta) = \exp(-\eta)$ and power-law decay of relevance ($\alpha_R = 1$) and activity ($\alpha_A = 0.4$); the other simulations details are the same described in the MM section of the main text. The artificial dataset calibrated on the APS data is grown using the RM with $\rho(\eta) = \exp(-\eta)$ and power law decay of relevance ($\alpha_R = 1.4$); all outgoing links of a node are created when the node enters the system and the outdegree distribution is $\rho(k) = 8.33\,\exp(-0.12\,k)$ as in the APS data (see Figure S4).

S4 Measuring empirical relevance in real and artificial data

Since fitness values are not known in real data, we use the total relevance defined by Eq. (3) as an estimator of node fitness. As shown for the RM in [1], total relevance and fitness are closely connected and both provide information about the perceived importance of a node. However, the direct use of Eq. (3) poses a problem in artificial data. In our numerical simulations, a constant number of link are added to the system at each time step. The factor $\sum_j k_j^{in}(t)$ on the right side of Eq. (3) consequently grows linearly with simulation time and, as a result, the total relevance computed with Eq. (3) is biased towards recent nodes. This issue does not occur in real data where both $L(t, \Delta t)$ and $\sum_j k_j^{in}$ grow with time. To avoid this bias, we omit the factor $\sum_j k_j^{in}$ when computing relevance in real data and use the following definition

$$\tilde{r}_i(t; \Delta t) = \frac{n_i(t; \Delta t)}{k_i^{in}(t; \Delta t)}. \tag{S3}$$

The corresponding definition of total relevance for model data is $T_i(t; \Delta t) := \sum_t \tilde{r}_i(t; \Delta t)$; we use $\Delta t = 20$. This quantity is used in Figure 5 in the main text to compare the rankings by indegree and PageRank on calibrated artificial data. In these simulations, we find $r(T, \eta) = 0.71$ and $r(T, \eta) = 0.65$ for the RM calibrated on the Digg.com and APS dataset, respectively. High values of the correlation between T and η confirm that total relevance is a suitable estimator of a node's intrinsic fitness.

S5 Production of Figure 1 in the main text

To produce Figure 1, we start from a network with two nodes: node 0 and node 1, and one link between them (from 1 to 0). The final network consists of $N = 40$ nodes and is grown up according to the RM. Node fitness is drawn from the exponentia distribution $\rho(\eta) = \exp(-\eta)$. Node relevance decays exponentially with $f_R(t) = (0.4)^t$. At each simulation step, a new node is added to the system and connected to an existing node according to Eq. 1 in the main text. Consequently, one new link is created among the existing nodes (so-called internal link). While the target node is again chosen according to Eq. 1, the starting node is chosen at random from the existing nodes which corresponds to constant node activity.

S6 Assessing the uncertainty of results

Non-parametric bootstrap is a statistical method to estimate the error on quantities measured in real data [?, 3]. To estimate the errors of the correlation coefficients $r(k^{in}, T)$ and $r(p, T)$ for a real dataset, we create new datasets by resampling with repetition from the given dataset. Since a resampled dataset can in principle contain multiple links between a pair of nodes, we compute PageRank on the resampled data using the generalized formula

$$p_i^{(t+1)} = c \sum_j M_{ji} \frac{p_j^{(t)}}{k_j^{out}} + \frac{1-c}{N}, \tag{S4}$$

where M_{ji} is the number of directed links from j to i (and correspondingly $k_j^{out} = \sum_i M_{ji}$). The correlation values of interest can be computed for resampled dataset. The standard deviation of these results over datasets then characterizes the uncertainty of the original correlation values. For the Digg.com data, we obtain $r(k^{in}, T) = 0.330 \pm 0.003$ and $r(p, T) = 0.224 \pm 0.003$ which means that the uncertainty is small and insignificant in comparison with the absolute differences between the correlation values. Results for the APS data lead to the same conclusion.

For the results obtained with calibrated simulations, we estimate their uncertainty by analyzing several model realizations and evaluating the standard error of the mean for a quantity of interest. Using 50 model realisations, we find $r(k^{in}, T) = 0.444 \pm 0.002$ and $r(p, T) = 0.411 \pm 0.002$ which has the same implications as before: the results' uncertainties are substantially smaller than the absolute difference and thus unsignificant. Results for the APS data lead to the same conclusion.

S7 Relation between outdegree and activity

Eq. (2), which governs the creation of outgoing links, does not contain the preferential attachment mechanism and as a consequence the final outdegree is determined only by node activity A_i. When activity decay f_A is sufficiently fast to allow the normalisation factor of Π^{out} to converge, the asymptotic solution in the continuum approximation [4] reads

$$\overline{k_i^{out}(t)} = 1 + m\, A_i \, \frac{\int_{\tau_i}^{t} dt'\, f_A(t' - \tau_i)}{\Omega_\infty^{out}}, \tag{S5}$$

where τ_i is the time at which node i has entered the system and $\Omega_\infty^{out} = \lim_{t\to\infty} \sum_i A_i\, f_A(t - \tau_i) < \infty$. When activity decay is absent, this has an asymptotic solution

$$\overline{k_i^{out}(t)} = 1 + m\, \frac{A_i}{\overline{a}}\, \log\left(\frac{t}{\tau_i}\right) \tag{S6}$$

where $\overline{a}$ is the average node activity. The outdegree distribution is consequently determined mainly by the activity distribution $\rho(A)$.

S8 Supplementary figures: Analysis of real data

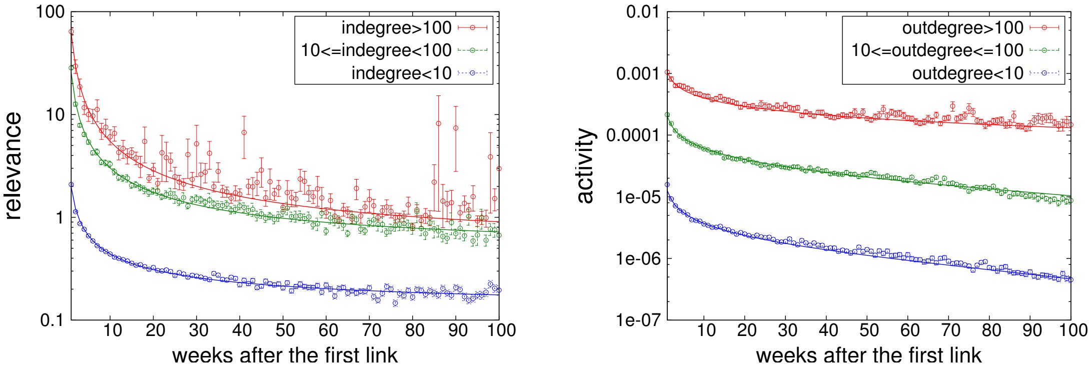

Figure S1: **Temporal decay of the average relevance $r(t)$ (left panel) and activity $a(t)$ (right panel) in Digg.com social network** (2006-2008, $\Delta t = 1$ week, color online). Symbols represent the average relevance and activity of nodes belonging to the same age group, error bars represent the errors of the mean, lines represent the fits described in Section S1.

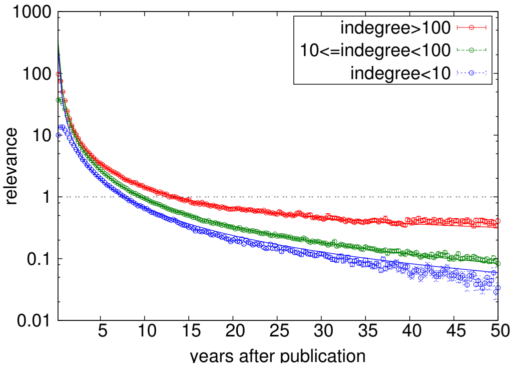

Figure S2: **Temporal decay of the average relevance $r(t)$ in the APS dataset** (1893-2009, $\Delta t = 91$ days, color online). Symbols represent the average relevance of nodes belonging to the same age group, error bars represent the error of the mean, lines represent the fits described in Section S2. The initial non-monotonous part of the relavance profile is ignored by the fitting procedure and consequently the fitted curves do not match the points corresponding to the first few years after publication.

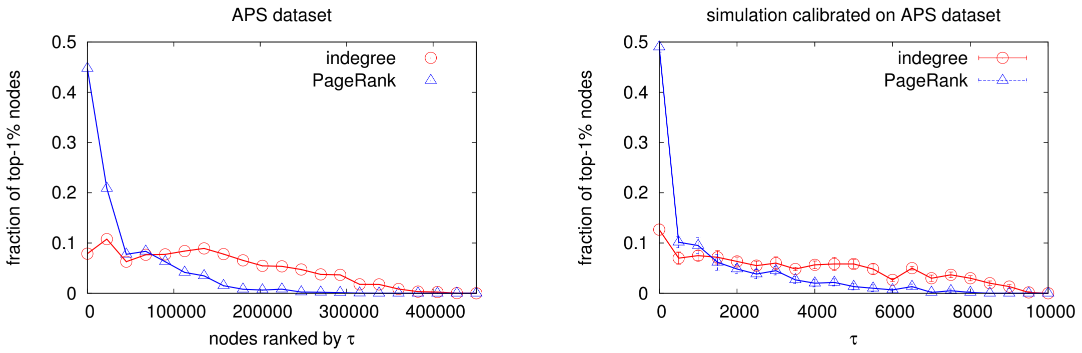

Figure S3: **Age distribution of the top 1% nodes in the ranking, in the APS data (left panel) and in the corresponding calibrated simulation (right panel)** (color online). On the x-axis, nodes are ordered by age (oldest on the left, youngest on the right). We see that PageRank is more biased towards old nodes than indegree. Results obtained on the calibrated model (see Table 2 for model parameters) agree well with those obtained on the real data.

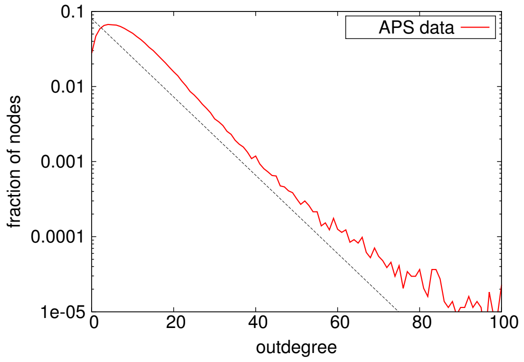

Figure S4: **Outdegree distribution in the APS dataset.** The distribution is narrow and is well approximated by $\rho(k^{out}) \sim \exp(-0.12\, k^{out})$ for $k^{out} > 5$ (black line). We use this distribution to generate the number of outgoing links created by a new node in the simulations calibrated on the APS dataset.

S9 Supplementary figures: Numerical simulations

For the following figures, unless stated otherwise, the models' settings are:

- RM: $N = 10,000$, $\rho(\eta) = \exp(-\eta)$, $f_R(t) = \exp(-t/\theta_R)$, $f_A(t) = \exp(-t/\theta_A)$.
- EFM: $N = 10,000$, $H = 250$, $f_R(t) = \exp(-t/\theta_R)$, $f_A(t) = \exp(-t/\theta_A)$.

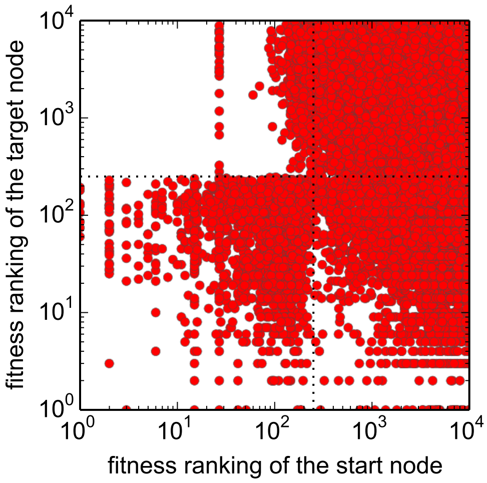

Figure S5: **Linking pattern in the extended fitness model (EFM).** Each symbol corresponds to a link from a start node whose rank according to fitness is shown on the horizontal axis to a target node whose rank according to fitness is shown on the vertical axis. The EFM parameters used to generate the network are $N = 10,000$, $\theta_R = 500$, $\theta_A = 10000$, $H = 250$. The dotted lines mark the rank position 250 that separates high-fitness nodes (whose fitness η is uniformly distributed in the range $[10^{-5}, 1]$) and low-fitness nodes (whose η is uniformly distributed in the range $[0, 10^{-5})$). We see that the EFM model produces networks where high-fitness nodes are typically pointed by other high-fitness nodes, thus creating a suitable setting for the PageRank algorithm.

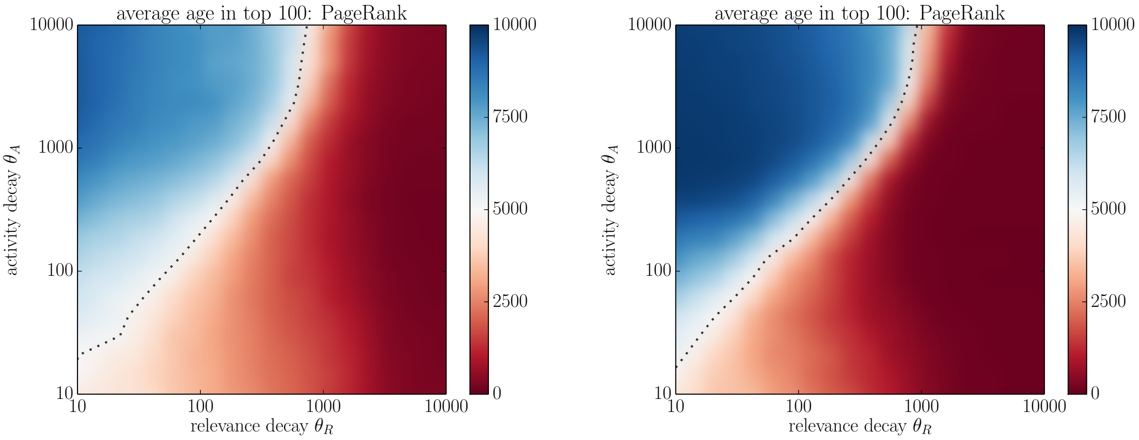

Figure S6: **Average birth time τ of the top 1% of nodes as ranked by PageRank in the RM (left) and in the EFM (right), color online.** When relevance decay is faster than activity decay (upper-left corners of the plots), PageRank is biased towards recent nodes (blue-shaded areas) in both models. When the opposite is true (lower-right corners of the plots), PageRank is biased towards old nodes (red-shaded areas). Between the two biased regions, there is a nearly-diagonal contour (marked with the dotted line) where the average age of top 100 nodes is $N/2 = 5000$ which means that the top 100 PageRank positions show no bias towards recent or old nodes. We can conclude that PageRank is not biased only when the timescales of relevance and activity decay are in accord. Results are averaged over 6 model realizations.

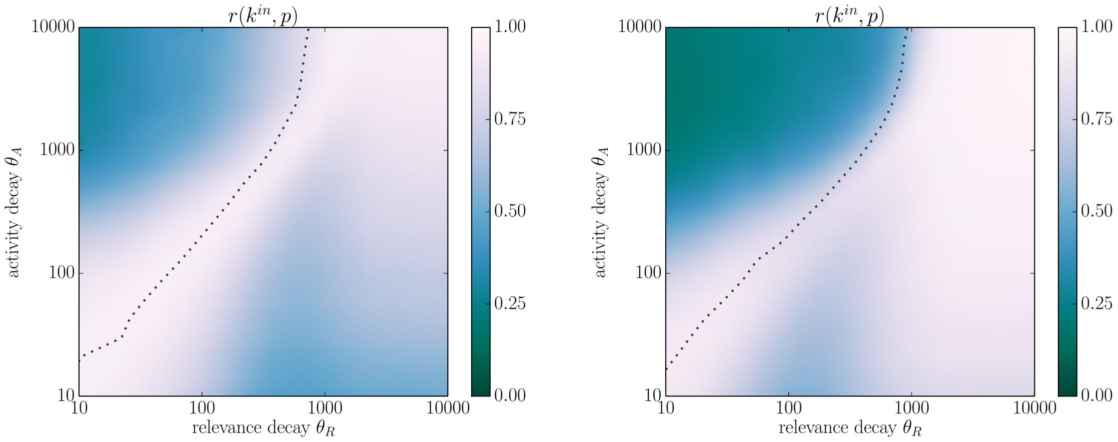

Figure S7: **Indegree-PageRank correlation in the RM (left) and EFM (right).** The dotted lines represent the zero-bias contours from Figure S6. When the timescales of relevance and activity decay mismatch (upper-left and lower-right corners of the heatmaps), indegree-PageRank correlation is weak due to the time bias of PageRank. This correlation is maximal near the contour where PageRank is not biased.

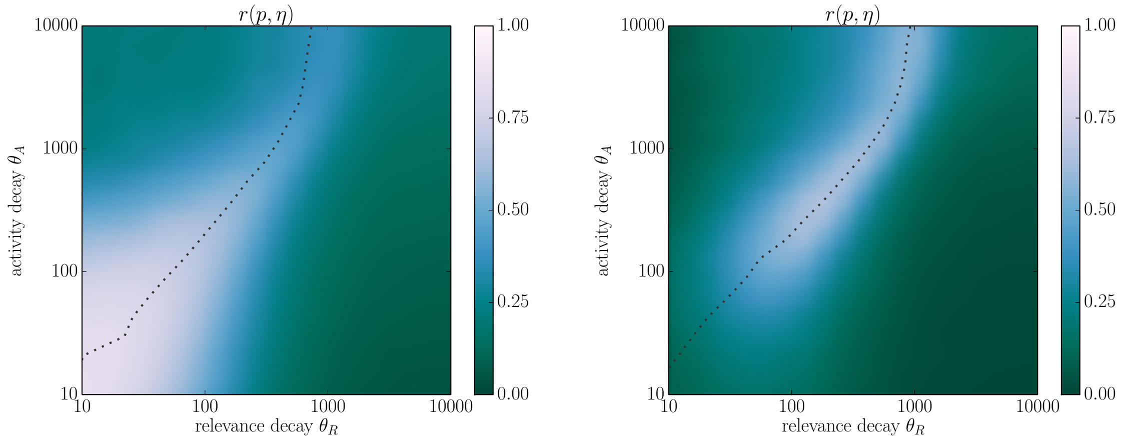

Figure S8: **Fitness-PageRank correlation in the RM (left) and EFM (right).** The dotted lines represent the zero-bias contours from Figure S6. When the timescales of relevance and activity decay mismatch (upper-left and lower-right corners of the heatmaps), fitness-PageRank correlation is weak due to the time bias of PageRank. PageRank performs best along the zero-bias contour. Note that while the global maximum of PageRank's performance for the RM occurs when both θ_A and θ_R are small (lower-left corner), the global maximum for the EFM is located in the center area. This happens because in the EFM, only a small fraction (5%) of nodes are sensitive to fitness; as a result, when activity and relevance decays are too fast, fluctuations damage the capability of indegree and PageRank to efficiently detect fitness. By contrast, in the RM all nodes are sensitive to fitness; for this reason, $\theta_R = 10$ and $\theta_A = 10$ are large enough to allow the system to significantly perceive fitness.

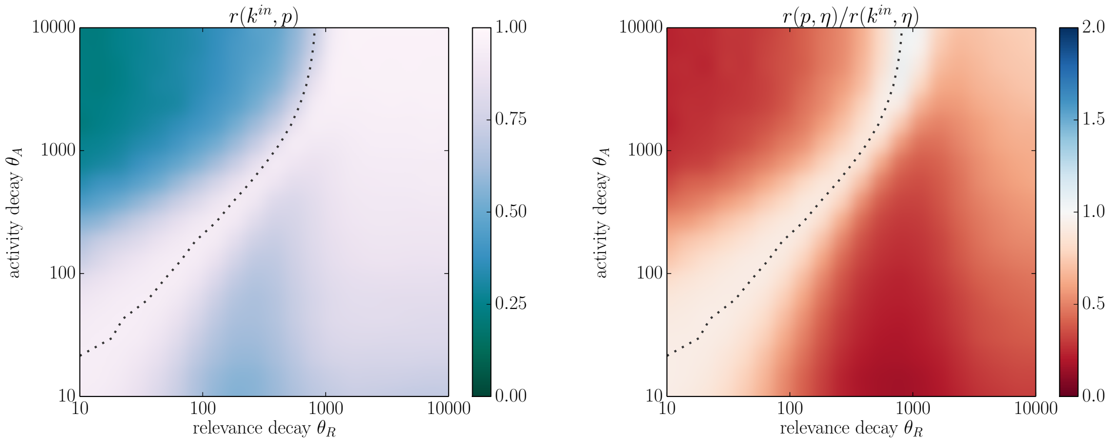

Figure S9: **Comparison between PageRank and indegree in the RM with uniform fitness distribution** (color online). Panels show the correlation $r(k^{in}, p)$ (left) and the correlation ratio $r(p, \eta)/r(k^{in}, \eta)$ (right) in the RM with node fitness η distributed uniformly in $[0, 1]$ (as opposed to the original exponential distribution). The dotted lines represent the PageRank's zero-bias contour for this model (bias is again evaluated on the basis of the average age in top 100 positions of the Pagerank ranking). Results show no significant differences from the results on the RM with an exponential fitness distribution (see the left panel in Figure S7 and the right panel in Figure 3 in the main text).

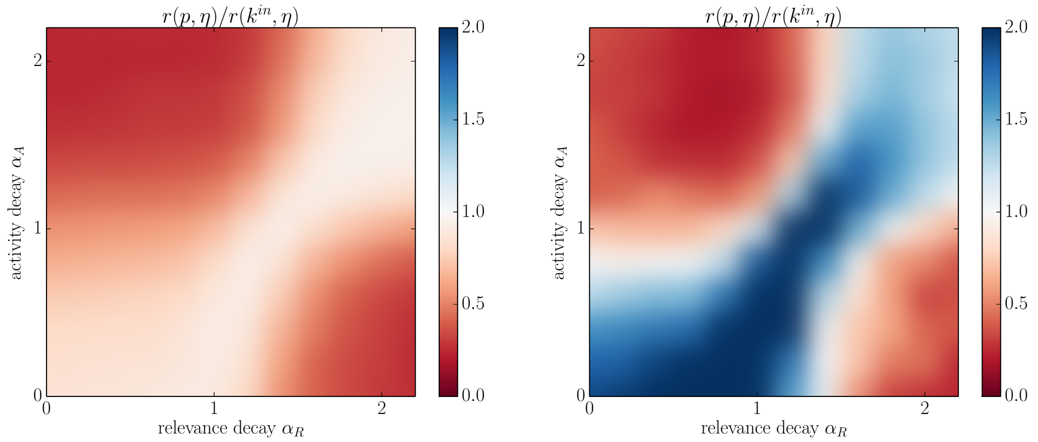

Figure S10: **Comparison between PageRank and indegree in the RM (left) and EFM (right) with power-law aging** (color online). We see here qualitatively the same behaviour as reported in Figures 3 and 4 in the main text where exponential aging is assumed. Two regions where PageRank fails are again present: one in which relevance decays faster than activity (lower-right corner) and one where the opposite is true (upper-left corner). In both the RM and the EFM, the regions where PageRank is not heavily outperformed by indegree include also the (α_R, α_A) values found in Digg.com data analysis (see Table 1). For simplicity, we focus on the parameter values $(\alpha_R, \alpha_A) = (1, 0.4)$, even if values in Table 1 are slightly different and dependent node degree group. In the RM, PageRank performance in ranking nodes by fitness is not far from that of indegree [$(1, 0.4)$ lies in the white-shaded area of left panel], which explains why indegree and PageRank performances in ranking nodes by relevance are close to each other in Digg.com dataset (see Fig. 5 of the main text). In the EFM, PageRank even outperforms indegree for these parameter values [$(1, 0.4)$ lies in the blue-shaded area of right panel], which illustrates the main difference between the favorable and unfavorable parameter regions for PageRank: when PageRank is biased because of temporal effects, it fails both in the RM and in the EFM; by contrast, when PageRank is not biased, then its performance with respect to indegree depends on the growth rule of the system and can benefit from a suitable model such as the EFM.

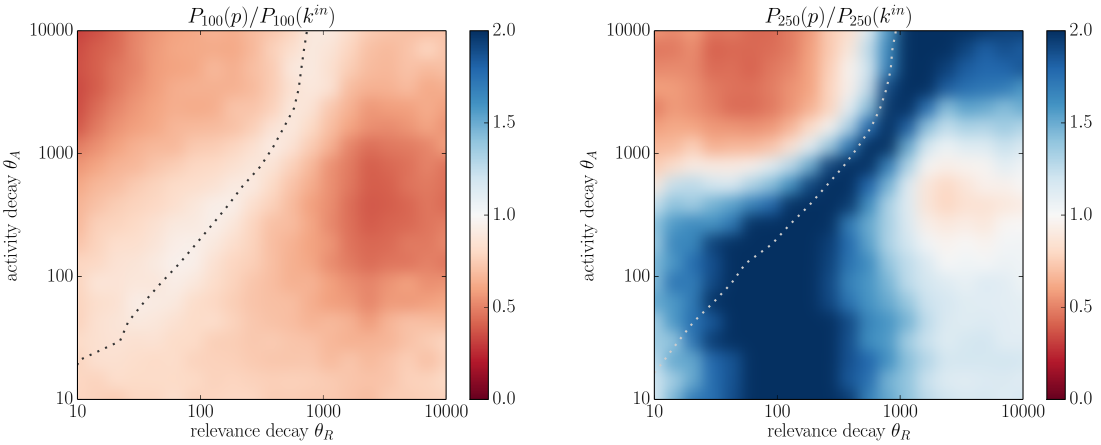

Figure S11: **Comparison between PageRank and indegree in the RM (left) and EFM (right) using precision** (color online). Precision $P_X(s)$ of the ranking by score s is the average number of nodes that are among the top X places of the fitness ranking that are at the same time at the top X places of the ranking by score s. The dotted lines represent the zero-bias contours from Figure S6. As for the score comparison on the basis of correlation with node fitness, PageRank's performance is again optimal along the contour of its zero time bias. In comparison with the correlation-based results, PageRank now lags less behind indegree in the region where relevance decays slowly and activity decays quickly (lower-right corner). However, both scores perform badly in this region (for the EFM, for example, $P_{250}(p) = 0.16$ in the lower-right corner as opposed to the best-achieved precision $P_{250}(p) = 0.69$ when $\theta_R = 183$ and $\theta_A = 263$).

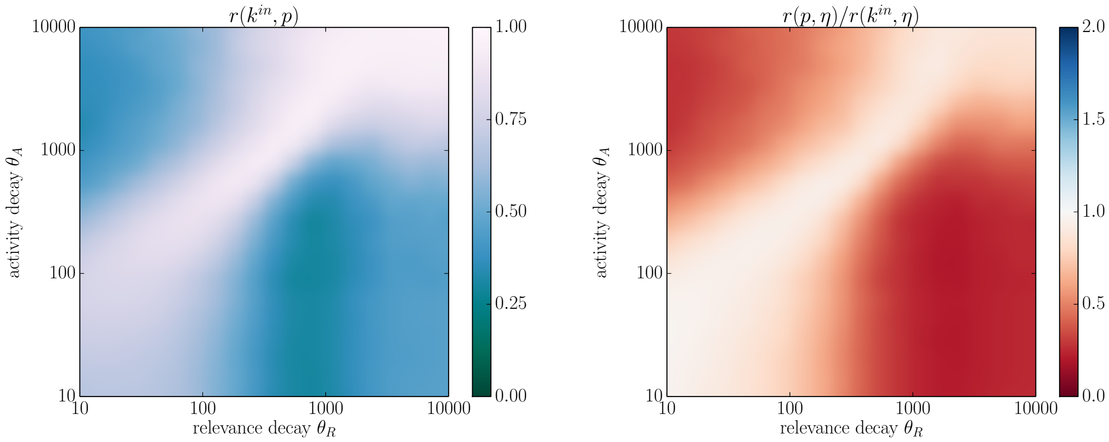

Figure S12: **Comparison between PageRank and indegree in the RM with accelerated growth** (color online). We grow networks according to the RM with the same parameters as before except that in simulation step t, $m(t) = 20\,t/N$ existing nodes are sequentially chosen and create one link each. In this way, the rate at which links are created in the network grows linearly with network size. This aims to represent real systems where the total node activity is not normalized but rather grows with the number of existing nodes. At the same time, the resulting number of links in the network is the same as before when $m = 10$ nodes were chosen and created one link each which makes the present model comparable with the original one. Panels show the correlation $r(k^{in}, p)$ (left) and the correlation ratio $r(p, \eta)/r(k^{in}, \eta)$ (right). The behaviour is again qualitatively similar to that found for uniform growth (see Figures 3 and S7) except that the lower-right region where PageRank fails is not even more pronounced. This further demonstrates the generality of our observations.

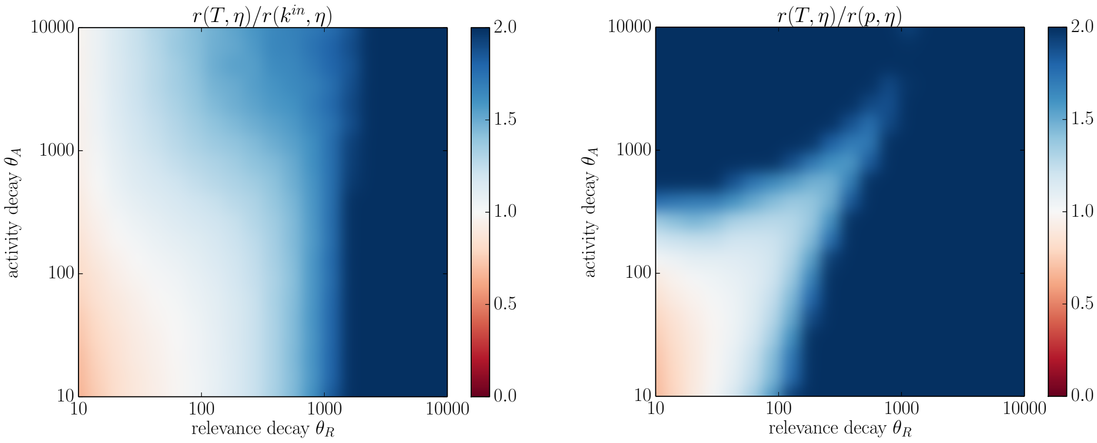

Figure S13: **Comparison of total relevance with indegree (left) and PageRank (right) in the RM** (color online). Total relevance outperforms indegree k^{in} and PageRank p in ranking nodes by fitness η for a broad range of model parameter. The performance ratios are particularly large for slow decay of relevance: for instance, the maximum value of $r(T,\eta)/r(k^{in},\eta)$ is found for $(\theta_R,\theta_A) = (4832, 14)$ $[r(T,\eta)/r(k^{in},\eta) = 2.83]$, and the maximum value of $r(T,\eta)/r(p,\eta)$ is found for $(\theta_R,\theta_A) = (3359, 10)$ $[r(T,\eta)/r(p,\eta) = 12.49]$. These findings show that total relevance is highly informative on node fitness and might motivate the study of the ranking by total relevance in real data.

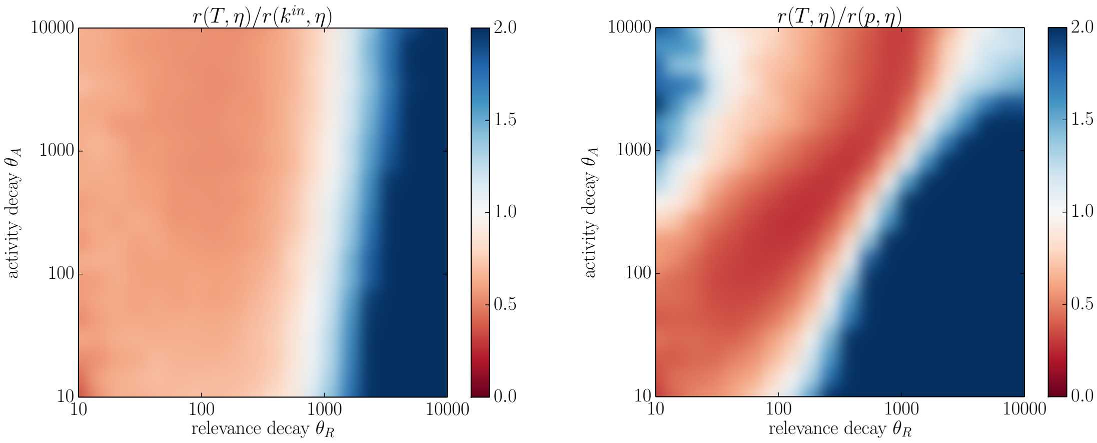

Figure S14: **Comparison of total relevance with indegree (left) and PageRank (right) in the EFM** (color online). In the data produced by the EFM, the parameter region where total relevance T outperforms indegree k^{in} and PageRank p in ranking nodes by fitness is smaller with respect to that observed for the RM. The performance ratio $r(T,\eta)/r(k^{in},\eta)$ becomes large when relevance decay is slow and, as a consequence, indegree is heavily biased towards old nodes; the maximum value of $r(T,\eta)/r(k^{in},\eta)$ is found for $(\theta_R,\theta_A) = (10000, 10)$ $[r(T,\eta)/r(k^{in},\eta) = 3.77]$. The maximum value of $r(T,\eta)/r(p,\eta)$ is found for $(\theta_R,\theta_A) = (10000, 10)$ $[r(T,\eta)/r(p,\eta) = 142.57$, with $r(T,\eta) = 0.172$ and $r(p,\eta) = 0.001]$. On the other hand, there are broad regions of parameter values where total relevance is outperformed by indegree and PageRank, which leaves the following question open: to what extent this failure of total relevance is due to the details of the model, such as the choice of $\rho(\eta)$ and the functional form of Π^{in}? Answering this question goes beyond the scope of this work.

References

[1] Medo, M., Cimini, G. & Gualdi, S. Temporal effects in the growth of networks. *Physical Review Letters* **107**, 238701 (2011).

[2] Perra, N., Gonçalves, B., Pastor-Satorras, R. & Vespignani, A. Activity driven modeling of time varying networks. *Scientific Reports* **2** (2012).

[3] Shalizi, C. The bootstrap. *American Scientist* **98**, 186–190 (2010).

[4] Newman, M. *Networks: an introduction* (Oxford University Press, 2010).
